# Evaluation of genetic association of neurodevelopment and neuroimmunological genes with antipsychotic treatment response in schizophrenia in Indian populations

**DOI:** 10.1002/mgg3.169

**Published:** 2015-08-09

**Authors:** Ajay Jajodia, Harpreet Kaur, Kalpana Kumari, Neha Kanojia, Meenal Gupta, Ruchi Baghel, Mamta Sood, Sanjeev Jain, Rakesh K. Chadda, Ritushree Kukreti

**Affiliations:** ^1^ Genomics and Molecular MedicineCSIR‐Institute of Genomics and Integrative BiologyMall RoadDelhi110007India; ^2^ Department of PsychiatryAll India Institute of Medical SciencesAnsari NagarNew Delhi110029India; ^3^ Molecular Genetic LaboratoryDepartment of PsychiatryNational Institute of Mental Health and Neuro SciencesHosur RoadBengaluru560029India

**Keywords:** Antipsychotic, meta‐analysis, neurodevelopment, pharmacogenomics, severity

## Abstract

Neurodevelopmental and neuroimmunological genes critically regulate antipsychotic treatment outcome. We report genetic associations of antipsychotic response in 742 schizophrenia patients from Indian populations of Indo‐European and Dravidian ancestry, segregated by disease severity. Meta‐analysis comparing the two populations identified *CCL2* [rs4795893: OR (95% CI) = 1.79 (1.27–2.52), *P *=* *7.62 × 10^−4^; rs4586: OR (95% CI) = 1.74 (1.24–2.43), *P *=* *1.13 × 10^−3^] and *GRIA4* [rs2513265: OR (95% CI) = 0.53 (0.36–0.78), *P *=* *1.44 × 10^−3^] in low severity group; and, *ADCY2* [rs1544938: OR (95% CI) = 0.36 (0.19–0.65), *P *=* *7.68 × 10^−4^] and *NRG1* [rs13250975, OR (95% CI) = 0.42 (0.23–0.79), *P *=* *6.81 × 10^−3^; rs17716295, OR (95% CI) = 1.78 (1.15–2.75), *P *=* *8.71 × 10^−3^] in high severity group, with incomplete response toward antipsychotics. To our knowledge, this is the first study to identify genetic polymorphisms associated with the efficacy of antipsychotic treatment of schizophrenia patients from two major India populations.

## Introduction

Schizophrenia is a debilitating psychiatric illness, with an estimated heritability up to 80%. Determinants to the disease include both genetic and environmental risk factors. Interindividual variations are observed in differential response toward antipsychotic medication (Zandi and Judy 2010). Previously, importance of clinical and genetic factors on therapeutic outcome in patients with differential range of disease severity (Chen et al. 2009) and stages of illness (Emsley et al. 2006) has been highlighted. Disturbances in neurodevelopmental processes due to alteration in neurotransmitter signaling and involvement of neuroimmunological and genetic factors have been suggested in the pathophysiology of schizophrenia (Lieberman et al. 2005; Altamura et al. 2013). Pharmacogenomics presents the opportunity to discover genetic variants predictive of drug response. This may enable clinicians to choose the most optimal drug with less trial and error.

Antipsychotic medication aims to ameliorate a range of symptoms and reduce disease severity. Responsiveness to antipsychotic medication varies according to the stage of illness (Emsley et al. 2006) and differential range of disease severity (Chen et al. 2009). Discontinuation frequency of antipsychotic treatment is quite high due to the apparent lack of efficacy and intolerable side effects (Lieberman et al. 2005). Hence, early response toward medication is a predictor of long‐term response (Kinon et al. 2010). Therefore, it is important to investigate the role of clinical and genetic factors in the therapeutic outcome in patients with differential range of disease severity (Chen et al. 2009).

A number of genome‐wide association studies (GWASs), postmortems, and animal studies (Ikeda et al. 2010; McClay et al. 2011) have implicated the role of susceptibility genes and related neurodevelopment pathways in schizophrenia pathophysiology and treatment outcome (Banerjee et al. 2010; Iasevoli et al. 2014). Since antipsychotics are neurogenic in action, research has focused on the involvement of neurodevelopmental genes in antipsychotic response (Newton and Duman 2007). Reports have also suggested the role of immune‐related genes and immune alteration in schizophrenia (Schwarz et al. 2006). Abnormal levels of pro‐ and anti‐inflammatory cytokines have been detected in peripheral blood and cerebrospinal fluids of schizophrenia patients (Hope et al. 2009; Beumer et al. 2012). Genetic variants of interleukins have been intensively studied in schizophrenia (Paul‐Samojedny et al. 2010). Evidences from above studies provide the role of neurodevelopmental and immune abnormalities, in predicting response of antipsychotic treatment.

In the present study, we investigated polymorphisms in the genes involved in neuregulin signaling (Chen et al. 2006; Mei and Xiong 2008; Shamir et al. 2012), neuro‐active ligand receptor (Putnam et al. 2011; Adkins et al. 2012; Ayalew et al. 2012; Liu et al. 2013), and immune‐related genes (Schwarz et al. 2006; Hope et al. 2009) in 742 antipsychotic‐treated schizophrenia cases in two independent populations (North and South India), comparing complete‐ and incomplete responders within low and high disease severity cohorts.

## Material and Methods

### Subjects

Patients of either gender taking typical and atypical antipsychotics irrespective of treatment history were recruited from the Clinical Services of Outpatients Department of Psychiatry Services, All India Institute of Medical Sciences, New Delhi and the National Institute of Mental Health and Allied Sciences, Bengaluru, respectively. Patients were diagnosed using the criteria described in the Diagnostic and Statistical Manual of Mental Disorders, Fourth Edition (DSM‐IV‐TR) by experienced psychiatrists. Patients were excluded if they had (1) past or family history of any other mental/neurological disorders; (2) drug abuse; (3) head injury; or (4) pregnancy. Detailed clinical and treatment history including age of onset (AOO), duration of illness (DOI), medication, relapse, noncompliance, and change in treatment were also documented during enrollment. The age at onset of schizophrenia was defined as the age at first appearance of psychotic symptoms. This was a cross sectional and naturalistic follow‐up study and patients were not controlled for their medications. Drugs were prescribed as usual in clinical practice and clinicians were blinded to genotypes of the patients. Further, patients were assessed by the Clinical Global Impressions Severity scores (CGI‐S) (Busner and Targum 2007), at the time of enrollment and reassessed after 3 months of antipsychotic treatment. Medication was as chosen by the clinicians and administered drugs included typical antipsychotics such as chlorpromazine (200–400 mg/day), fluphenazine (20–50 mg/day), and flupenthixol (20–40 mg/day); and atypical antipsychotics such as clozapine (25–400 mg/day), risperidone (2–10 mg/day), olanzapine (5–20 mg/day), ziprasidone (40–160 mg/day), quetiapine (1200 mg/day), aripiprazole (10–45 mg/day), amisulpride (100–600 mg/day), and levosulpride (200 mg/day) or combination of two or more antipsychotic (atypical/typical) drugs. Antipsychotic doses to chlorpromazine equivalent are provided (Table S1) (Woods 2003). Complete medical history, medication, and patient follow‐up records were maintained at both the collection sites. Additionally, 401 (South India) and 385 (North India) healthy volunteers were recruited to investigate population stratification.

The first component of CGI, which measures severity of illness (CGI‐S), was used to classify patients into low severity group (LSG; baseline CGI‐S ≤ 3) and high severity group (HSG; baseline CGI‐S ≥ 4). The second component of CGI, measuring global improvement after medication (CGI‐I), was used to classify patients as complete responders (CGI‐I < 2 or a drop of two after treatment) and incomplete responders (CGI‐I ≥ 3) (Gupta et al. 2012; Kaur et al. 2014). It is expected that severely ill patients have poorer treatment outcome, especially if they do not respond to several months of treatment and their symptoms may become more severe if they are not responding. Existing literature supports evaluation period of 3 months to understand antipsychotic response (Lehman et al. 2004; Meltzer 2008). Thus for the present study, patients were followed for 3 months during which they were treated with antipsychotic drugs (Tybura et al. 2012).

### Ethics statement

Informed consent was obtained for all participants. For participants with compromised ability, consent was taken from respective family member. The study was approved by the Institutional Ethics Committee and Review Boards of participating Hospitals.

### Population stratification

A major concern of population‐based association is stratification in the sample pool which can serve as a potential confounder and may lead to false positives. To prevent this, participants were recruited from the same geographic regions and of same ethnicity. In addition, 401 and 385 healthy volunteers from South India and North India, respectively, were recruited to assess the possibility of population stratification. Exclusion criteria for controls individuals were any history of psychiatric illness or having family history of schizophrenia or other neurological disorders. In addition, we genotyped 552 neutral markers for testing the homogeneity in sample pool. These markers were selected from 4991 neutral markers form Affymetrix 50K data set (Jha et al. 2012). Population structure was investigated by Principal Component (PC) analysis performed for a set of neutral markers with Eigenstrat (Price et al. 2006). Additionally, stratification test was performed by STRUCTURE 2.1 (Pritchard et al. 2000). We assumed *K* = 2 and model parameters were burn‐in period, number of Markov Chain Monte Carlo (MCMC) repeats after burn‐in, and iterations as 500, 5000 and 1000, respectively, for Structured Population Association Test (STRAT) program.

### Variant selection

We utilized a custom set of 1536 SNPs from candidate genes of NRG1‐ERBB signaling pathway, neuroactive ligand–receptor interaction, glutamate signaling, and a follow‐up from previous GWAS (Need et al. 2009; Schizophrenia Psychiatric Genome‐Wide Association Study 2011; Shi et al. 2011). Variants located in the coding region (synonymous and nonsynonymous), noncoding region (5′ and 3′ untranslated region of the gene spanning regulatory regions), splice sites, and enhancer or silencer elements were specifically prioritized for genotyping. The selected variants were screened for their possible regulatory effect by utilizing RegulomeDb and Haploreg (Boyle et al. 2012; Ward and Kellis 2012).

### Genotyping

Genotyping was performed using a customized array of Illumina Genomestation using GoldenGate technology according to the manufacturer's guidelines. Eighty‐eight DNA samples served as duplicates for quality control measures of clustering and reproducibility. Primary genotyping data analyses were executed with GenomeStudio software and followed by visual inspection and assessment of data quality and clustering. Additionally, to rule out any genotyping error 15% of the total samples were regenotyped randomly SNP genotyping platform–primer extension reaction followed by using MALDI‐TOF mass spectrometry platform (Sequenom™, Inc., SanDiego, CA).

### Genotype–phenotype association analyses

GenomeStudio Genotyping Module v1.0 was used to analyze SNPs of the Illumina custom panel for DNA sample validation. Quality control assessments were performed with PLINK 1.07 (Purcell et al. 2007). Genotyping and quality control measures were applied as previously described (Jajodia et al. 2015). Briefly, loci having poorly defined clusters, GenTrain score <0.6, more than 60% genotyping failure, excessive missingness, and Mendelian and replication errors were excluded. Hardy–Weinberg Equilibrium (HWE) was assessed for all the SNPs and a threshold of significance was *P* = 0.0001 in controls. SNPs with minor allele frequency (MAF) <1% and nonpolymorphic nature were not included in further analysis. Genotyping concordance was above 98% for Illumina and ~94.6% for Sequenom platform.

A study by researchers in Indian Genome Variation Consortium has documented a high level of population structure and has shown the presence of north and south gradient in Indian population (Indian Genome Variation, 2008). The two studied populations are ancestrally different. So, therefore it is not suggestive to pool the data from two populations. This may result in spurious association due to systematic ancestry differences in two populations. Hence, we have performed analysis separately for both the populations. Genomic inflation was controlled by utilizing first two principal components (Jajodia et al. 2015). Meta‐analysis was performed between complete‐ and incomplete responders within each severity group using adjusted *P*‐values of treatment response association analysis using PLINK 1.07. Heterogeneity was evaluated with the Cochran Q statistic test in the contributing data sets. Odds ratios (ORs) and 95% confidence intervals (CIs) were calculated using R script. Linkage Disequilibrium (LD) was estimated among SNPs by *r* ^2^ value for the genotype data of all markers in healthy controls using Tagger algorithm in Haploview program version 4.1 (Barrett et al. 2005). Identification of tagSNPs was performed using Tagger program.

## Results

### Demographic and clinical characteristics of subjects

The demographic characteristics of subjects eligible for genotype–phenotype analysis are summarized in Table 1. A total of 482 and 352 patients were recruited from NIMHANS and AIIMS, respectively; of which 366 patients were classified as complete responders and 376 were incomplete responders. All the quality control measure resulted in 436 SNPs in North Indian and 486 SNPs in South Indian samples available for homogeneity test. The stratification test showed homogeneity between all the analyzed samples from North Indian cohort (*χ* ^2^ = 413.41; df = 435; *P* = 0.799) and South Indian cohorts (*χ* ^2^ = 440.47; df = 485; *P* = 0.80). Similarly genomics inflation rate was below 1.05 in both the population pool after adjusting for first two principal components (Jajodia et al. 2015).

**Table 1:** Clinical features of schizophrenia patients stratified based on severity and completed 3‐month follow‐up from South (*n* = 419) and North (*n* = 323) India

Groups^a^ parameters	NIMHANS (South India)						AIIMS (North India)					
	Patients in low severity group (LSG) (*n* = 192; 45.6%)			Patients in high severity group (HSG) (*n* = 227; 54.4%)			Patients in low severity group (LSG) (*n* = 211; 65.32%)			Patients in high severity group (HSG) (*n* = 112; 34.68%)		
	CR *n* (%)	IR *n* (%)	*P*‐value^b^	CR *n* (%)	IR *n* (%)	*P*‐value^b^	CR *n* (%)	IR *n* (%)	*P*‐value^b^	CR *n* (%)	IR *n* (%)	*P*‐value^b^
Gender												
Male	80 (56.34)	30 (60)	0.76	39 (58.2)	101 (63.1)	0.58	69 (47.26)	39 (60)	0.11	9 (81.8)	53 (52.47)	0.12
Female	62 (43.66)	20 (40)		28 (41.8)	59 (36.9)		77 (52.74)	26 (40)		2 (18.2)	48 (47.53)	
Age (mean ± SD) years	29.95 ± 7.8	28.68 ± 7.76	0.32^c^	28.44 ± 6.24	29.35 ± 7.23	0.36^c^	32.06 ± 9.27	34.29 ± 8.63	0.10^c^	30.27 ± 7.97	33.10 ± 9.08	0.32^c^
Age at onset^d^												
Early	84 (59.1)	30 (60)	1	40 (59.7)	98 (61.25)	0.94	76 (52)	27 (41.53)	0.82	6 (55)	54 (53.46)	1
Late	58 (40.9)	20 (40)		27 (40.3)	62 (38.75)		70 (48)	38 (58.47)		5 (45)	47 (46.54)	
Duration of illness^e^												
Short	97 (68.3)	29 (58)	0.25	35 (52.23)	86 (53.75)	0.95	62 (42.46)	6 (9.23)	4.03E‐06	4 (36.37)	27 (26.73)	0.74
Long	45 (31.7)	21 (42)		32 (47.77)	74 (46.25)		84 (57.54)	59 (90.77)		7 (63.63)	74 (73.27)	

SD, standard deviation; CR, complete responder, IR, incomplete responder.

a Low severity ≤3 CGI‐S and High severity ≥4 CGI‐S.

b *P*‐values were calculated by Pearson's chi‐square test.

c *P*‐values were calculated by Pearson's Student's *t*‐test.

d Onset <25 years; late onset ≥25 years.

e Short duration <4 years; long duration ≥4 years.

### Drug response analyses

Antipsychotic response (phenotype) was compared between responders and nonresponder in the presence of genetic polymorphisms (genotype) using additive model regression methodology, adjusted for gender, age, disease onset age, disease duration, treatment type, and ancestry factors. Meta‐analysis was performed using adjusted *P*‐values of treatment response association analysis in the two populations, segregated on the basis of disease severity. A total of three SNPs demonstrated significant association in both the study groups individually and in combined analysis for both the severity groups (Tables 2 and 3). Finally, a total of 698 biallelic markers in North Indian samples and; 685 SNPs were made available for South Indian data set for drug response analyses.

**Table 2:** Single‐Nucleotide Polymorphisms‐based association study and meta‐analysis in low severity group

Chr	SNP	Location (bp)	Closest Gene	A1/A2	MAF^a^	Meta India		South India		North India	
						OR^b^ (95% CI)	*P*_meta_	OR (95% CI)	*P* c	OR (95% CI)	*P* c
17	rs4795893	32574448	*CCL2*	G/A	41%	1.794 (1.276–2.522)	0.000762	1.860 (1.133–3.053)	0.01398	1.736 (1.087–2.775)	0.02103
17	rs4586	32583269	*CCL2*	T/C	39%	1.742 (1.247–2.433)	0.001133	1.676 (1.034–2.719)	0.03602	1.803 (1.135–2.864)	0.01239
11	rs2513265	105423795	*GRIA4*	A/T	31%	0.534 (0.363–0.785)	0.001449	0.556 (0.315–0.980)	0.04257	0.516 (0.305–0.873)	0.01376

A1/A2, minor allele and major allele based on whole sample; BP, base position; CHR, chromosome (hg19); SNP single‐nucleotide polymorphism; OR, odds ratio; CI, confidence Interval; Meta, Meta‐analysis, *P*‐value of whole sample data.

a Frequency is the average minor allele frequency in the control sample set.

b OR was calculated on the basis of A1 in Meta India as reference.

c *P* values of association test while adjusted for gender, age, age at onset, drugs, and first two principal component assuming additive model in each of study group.

**Table 3:** Single‐Nucleotide Polymorphism based on association study and meta‐analysis in high severity group

Chr	SNP	Location (bp)	Closest Gene	A1/A2	MAF^a^	Meta India		South India		North India	
						OR^b^ (95% CI)	*P*_meta_	OR (95% CI)	*P* c	OR (95% CI)	*P* c
5	rs1544938	7634119	*ADCY2*	C/G	8%	0.36 (0.19–0.65)	0.000768	0.39 (0.2–0.75)	0.004702	0.2 (0.04–0.98)	0.04774
8	rs13250975	13250975	*NRG1*	G/A	9%	0.42 (0.23–0.79)	0.006818	0.5 (0.25–0.98)	0.04357	0.25 (0.06–0.9)	0.03457
8	rs17716295	17716295	*NRG1*	A/C	38%	1.78 (1.15–2.75)	0.008715	1.62 (1.02–2.56)	0.0382	3.93 (1.04–14.8)	0.04258

A1/A2, minor allele and major allele based on whole sample; BP, base position; CHR, chromosome (hg19); SNP, single‐nucleotide polymorphism; OR, odds ratio; CI, confidence interval; Meta, Meta‐analysis *P*‐value of whole sample data.

a Frequency is the average minor allele frequency in the control sample set.

b OR was calculated on the basis of A1 in Meta India as reference.

c *P* values of association test while adjusted for gender, age, age at onset, drugs, and first two principal component assuming additive model in each of study group.

### Low severity group

In Low Severity Group, drug response analysis identified two variants of *CCL2* to be significantly associated with antipsychotic treatment response (Table 2). SNP rs4795893 (G/A) is located on chromosome 17q11.2‐q12, approximately 8 kb upstream to *CCL2*. Frequency of G‐allele is approximately 41% in schizophrenia patients with antipsychotic treatment. The adjusted OR obtained in meta‐analysis of the two populations was 1.79 (95% CI: 1.27–2.52, *P *=* *7.62 × 10^−4^). SNP rs4586 (T/C) is located 973 bp upstream to the *CCL2* gene having T‐allele frequency of 39% in patients. In presence of this allele, the odds of achieving poor treatment outcome were 1.74 times higher with incomplete responders compared to complete responders (95% CI: 1.24–2.43, *P *=* *1.13 × 10^−3^).

The other SNP showing significant association with drug treatment was rs2513265 (A/T), located closer to the glutamate receptor ionotropic, *AMPA4* (*GRIA4*) gene. The combined OR was 0.53 (95% CI: 0.36*–*0.78, *P *=* *1.44 × 10^−3^), with A‐allele frequency of 31% in antipsychotic‐treated patients.

### High severity group

In High Severity Group (Table 3), drug response analysis indicated that the most significant variant associated with treatment outcome was rs1544938, located on chromosome 5p15.3 and in proximity to *ADCY2*. The SNP showed significant association with a protective effect and combined OR of 0.36 (95% CI: 0.19–0.65, *P *=* *7.68 × 10^−4^). The C allele is present in 8% of patients treated with antipsychotic drugs. The other two polymorphisms associated with antipsychotic response in HSG were rs13250975 and rs17716295. The former is located on chromosome 8p12 at 5′ region of the *NRG1* gene. The minor allele G was present at frequency of 9% in patients, showing protective effect and combined OR (95% CI) = 0.42 (0.23–0.79), *P *=* *6.81 × 10^−3^). The rs17716295 is present approximately 70 kb downstream of rs13250975 and presence of A‐allele was found in 38% of treated patients. The risk of poor outcome was approximately 1.78 times higher in presence of A‐allele in drug‐treated patients (OR (95% CI) = 1.78 (1.15–2.75), *P *=* *8.71 × 10^−3^).

## Discussion

Indian populations are an admixture of two ancestral groups – “Ancestral North India” (ANI) and “Ancestral South India” (ASI) (Reich et al. 2009). After their admixture, a very restricted genetic exchange between them resulted in a complex caste system. IGVC (Indian Genome Variation Consortium) has structured the Indian population on the basis of four major language families – Indo‐European, Dravidian, Austro‐Asiatic, and Tibeto‐Burman with a high level of genetic differentiation (Indian Genome Variation Consortium 2008). Genetic variability among populations may result in the differential response toward treatment. The present pharmacogenomics study aims to relate this genetic differentiation with the variability in antipsychotic response. To our knowledge, this study is the first to assess the effect of polymorphisms in neurodevelopmental and neuroimmunological genes associated with antipsychotic response of the two independent Indian populations.

Suggestive associations were observed for the SNPs rs4795893 and rs4586 with the gene *CCL2* in drug response in less severely ill schizophrenia patients. CCL2 (Chemokine C‐C motif ligand 2) is a small proinflammatory cytokine that plays a key role in recruiting monocytes. Levels of pro‐ and anti‐inflammatory cytokines, including IL‐2, IL‐6, IL‐10, and TNF‐*α* in peripheral blood and cerebrospinal fluid are reported to be altered in schizophrenia (Sacchetti et al. 2007; Potvin et al. 2008; Hope et al. 2009). We observed a moderate LD (r^2^ ≤ 0.80) between rs4586 and rs4795893 in the two populations. However, the reported LD between these SNPs was *r* ^2^ ≥ 0.95 in 1000 genome population (Ward and Kellis 2012). This phenomenon may be related to the differential demographic and cultural history of Indian population. A recent study has shown the role of *CCL2* variants (rs1024611, rs4586, and rs2857657) in developing resistance in risperidone‐treated schizophrenia patients of Chinese Han origin (Xiong et al. 2014). The present study shows rs1024611 in LD (*r* ^2^ = 0.72) with rs4586, that has been reported to affect the transcriptional activity and *CCL2* expression (Mundo et al. 2005). In silico analysis suggests that rs4586 (T/C) is localized in the EP300 transcription factor (TF)‐binding site (ENCODE data set). This TF shows efficient binding in the presence of C allele, which had a higher frequency in incomplete responders (Table S2). Moreover, *CCL2* is overexpressed in schizophrenia patients compared to healthy individuals (Beumer et al. 2012).

AMPA receptor variant, rs2513265 has been reported to be positively associated with iloperidone efficacy (Lavedan et al. 2009). AMPA is a non‐NMDA‐type ionotropic transmembrane receptor for glutamate that mediates fast synaptic transmission in the central nervous system. Glutamate is an excitatory neurotransmitter in the cortical circuitry and hence, glutamate synaptic dysfunction may plays a role in schizophrenia (Zoghbi and Bear 2012). Modulators of AMPA receptors, such as N‐biaryl (cyclo) alkyl‐2‐propanesulfonamides, have been found to be useful in the treatment of cognitive deficits associated with schizophrenia (Estep et al. 2008). AMPA receptor modulators have shown promising results while investigated treatment options for cognitive disorders (Zheng et al. 2011). Due to the complex nature of glutamate signaling in brain, genetic variants in glutamate receptors are likely contributors to the variability seen in symptom presentation and antipsychotic response (Bishop et al. 2005).

Among the genes of particular interest in severely in HSG, is Adenylyl Cyclase 2 (*ADCY2*), which is a class B member of Adenylyl cyclases. It is a membrane‐associated enzyme encoded by the *ADCY2* gene and catalyzes the formation of cyclic adenosine monophosphate (cAMP) from adenosine triphosphate (ATP), where cAMP is a secondary messenger involved in various signal transduction pathways. ADCY2 is an enzyme typically expressed in the human brain. Several neurotransmitter receptors are G protein–coupled receptors (GPCRs) that regulate the activity of ADCY. Thus, ADCY2 may be involved in antipsychotic action and bipolar disorder (Fribourg et al. 2011; Muhleisen et al. 2014; Xu et al. 2014).

Variant from Neuregulin (*NRG1*) genes in high severity group shows promising results in both populations. NRG1 interacts extensively with GABAergic, glutamatergic, and dopaminergic neurotransmission system and is a potential target for new antipsychotics (Deng et al. 2013). Moreover, antipsychotic treatment rescues some of the abnormal behavior in animals with compromised NRG1‐ERBB4 signaling (Moghaddam 2003; Bjarnadottir et al. 2007; Barros et al. 2009). Several case–control association studies in various ethnic groups have supported NRG1 as a candidate gene for schizophrenia treatment (Stefansson et al. 2003; Petryshen et al. 2005; Shiota et al. 2008; Ng et al. 2009). In support, our case–control association analysis for the variants associated with treatment response in both the populations shows rs13250975 to be associated with susceptibility to schizophrenia (Jajodia et al. 2015).

Antipsychotics, both typical and atypical, increase neurogenesis and proliferation of nonneuronal cells in subventricular and subgranular zone, respectively (Newton and Duman 2007). Understanding the signaling cascade and molecular partners involved in antipsychotic‐induced neurogenesis can help appreciate the therapeutic effects. There could be an overlap between pathways involved in disease progression and treatment response, as shown for dopamine and serotonin receptors (Arranz and de Leon 2007). As a fresh perspective, the polymorphisms in neurodevelopmental and neuroimmunological genes identified in our study should be considered as important players in explaining the efficacy of antipsychotics and disease mechanism.

## Conclusion

We have identified several genetic polymorphisms associated with the efficacy of antipsychotic treatment in schizophrenia patients across Northern and Southern India, two major linguistic groups of the country. Although, our findings do not withstand test for multiple comparison, result from meta‐analysis shows a same direction of effect in both ancestrally different populations. There is a need to conduct large‐scale pharmacogenomic studies in different ethnic groups to validate our findings. We have validated several established evidences for the association of genetic polymorphisms with treatment response of antipsychotic agents. Simultaneously, we tried to introduce the Indian perspective and added several new associations. The findings of our study show the importance of pharmacogenomics research across transethnic groups of India.

## Conflict of Interest

None declared.

## Supporting information


**Table S1.** Chlorpromazine (100 mg/day) equivalent doses for antipsychotics prescribed in present study.

**Table S2.** In silico functional analysis of associated SNP in antipsychotic treatment.
