# Supplementary material for: Evaluation of genetic association of neurodevelopment and neuroimmunological genes with antipsychotic treatment response in schizophrenia in Indian populations
Source: Mol Genet Genomic Med. 2015 Aug 9;4(1):18–27. doi: 10.1002/mgg3.169 (PMC4707035; doi:10.1002/mgg3.169)
Supplement: Supplementary file 1 — Table S1. Chlorpromazine (100 mg/day) equivalent doses for antipsychotics prescribed in present study. [file MGG3-4-018-s001.docx]

Table S1: Chlorpromazine (100mg/day) equivalent doses for antipsychotics prescribed in present study

| **Generic Name of Antipsychotics** | **Equivalent dose (mg/day)** |
| --- | --- |
| Risperidone | 2 |
| Olanzapine | 5 |
| Clozapine | 100 |
| Ziprasidone | 60 |
| Quetiapine | 75 |
| Aripiprazole | 7.5 |
| Amisulpride | 100 |
