# Supplementary material for: Evaluation of genetic association of neurodevelopment and neuroimmunological genes with antipsychotic treatment response in schizophrenia in Indian populations
Source: Mol Genet Genomic Med. 2015 Aug 9;4(1):18–27. doi: 10.1002/mgg3.169 (PMC4707035; doi:10.1002/mgg3.169)
Supplement: Supplementary file 2 — Table S2. In silico functional analysis of associated SNP in antipsychotic treatment. [file MGG3-4-018-s002.docx]

| Table S2 *:In silico* functional analysis of associated SNP in anti-psychotic treatment | | | | | | |
| --- | --- | --- | --- | --- | --- | --- |
| **Chr** | **SNP** | **Pos(hg19)** | **Closest gene** | **Binding Affinity scores** | | |
|  |  |  |  | **Position weight matrices** | **Reference** | **Alternate** |
| 17 | rs4586 | 32583269 | CCL2 | Regulatory motif altered | T | C |
|  |  |  |  | **EP300** | **-0.1** | **10.9** |
|  |  |  |  | SMC3_DISC1 | 10.3 | 9.7 |
|  |  |  |  | Myf_4 | 12.6 | 11.9 |
|  |  |  |  | Myf_3 | 10.4 | 12.1 |
|  |  |  |  | CTCF_disc10 | 14.5 | 13.7 |
|  |  |  |  |  |  |  |
| 11 | rs2513265 | 105423795 | *GRIA4* | **Regulatory motif altered** | T | A |
|  |  |  |  | Smad_1 | 9.2 | 10.2 |
|  |  |  |  |  |  |  |
|  |  |  |  |  |  |  |
| 8 | rs13250975 | 32392809 | *NRG1* | **Regulatory motif altered** | A | G |
|  |  |  |  | Irx | 15.1 | 8.8 |
|  |  |  |  |  |  |  |
| 8 | rs17716295 | 32317917 | *NRG1* | **Regulatory motif altered** | C | A |
|  |  |  |  | Foxa_disc3 | 11.8 | -0.1 |
|  |  |  |  | COMP1 | 15.2 | 4.8 |
|  |  |  |  | Mrg_2 | 10.7 | 6.7 |
|  |  |  |  | STAT_known15 | 14.2 | 3.3 |
| 5 | rs1544938 | 7634232 | *ADCY2* | **Regulatory motif altered** | G | C |
|  |  |  |  | MZF1::1-4_1 | 11 | 10 |
|  |  |  |  | MZF1::1-4_2 | 12.2 | 8.4 |
|  |  |  |  | Pax-4_4 | 12 | 10.8 |
|  |  |  |  | SP2_disc2 | 5.1 | 11.8 |
|  |  |  |  | Spz1_1 | 11.7 | 12.7 |
|  |  |  |  | UF1H3BETA | -1.6 | -13.2 |
|  |  |  |  | Zfp740 | 11.8 | 7.6 |
|  |  |  |  |  |  |  |
